# Reliability of hip joint position sense tests using a clinically applicable measurement tool in elderly participants with unilateral hip osteoarthritis

**DOI:** 10.1038/s41598-021-04288-3

**Published:** 2022-01-10

**Authors:** Ravi Shankar Reddy, Jaya Shanker Tedla, Mastour Saeed Alshahrani, Faisal Asiri, Venkata Nagaraj Kakaraparthi, Paul Silvian Samuel, Praveen Kumar Kandakurti

**Affiliations:** 1 Department of Medical Rehabilitation Sciences, College of Applied Medical Sciences, King Khalid University, Abha, Saudi Arabia; 2 College of Health Sciences, Gulf Medical University, Ajman, United Arab Emirates

**Keywords:** Musculoskeletal system, Rheumatic diseases

## Abstract

Hip joint proprioception is vital in maintaining posture and stability in elderly individuals. Examining hip joint position sense (JPS) using reliable tools is important in contemporary clinical practice. The objective of this study is to evaluate the intra-rater and inter-rater reliability of hip JPS tests using a clinically applicable measurement tool in elderly individuals with unilateral hip osteoarthritis (OA). Sixty-two individuals (mean age = 67.5 years) diagnosed with unilateral hip OA participated in this study. The JPS tests were evaluated using a digital inclinometer in hip flexion and abduction directions. The absolute difference between target and reproduced angle (repositioning error) in degrees was taken to measure JPS accuracy. The intraclass correlation coefficient (ICC (2.k), was used to assess the reliability. The Intra rater-reliability for hip JPS tests showed *very good* agreement in the lying position (hip flexion-ICC = 0.88–0.92; standard error of measurement (SEM) = 0.06–0.07, hip abduction-ICC = 0.89–0.91; SEM = 0.06–0.07) and *good* agreement in the standing position (hip flexion-ICC = 0.69–0.72; SEM = 0.07, hip abduction-ICC = 0.66–0.69; SEM = 0.06–0.08). Likewise, inter-rater reliability for hip JPS tests demonstrated *very good* agreement in the lying position (hip flexion-ICC = 0.87–0.89; SEM = 0.06–0.07, hip abduction-ICC = 0.87–0.91; SEM = 0.07) and *good* agreement in the standing position (hip flexion-ICC = 0.64–0.66; SEM = 0.08, hip abduction-ICC = 0.60–0.72; SEM = 0.06–0.09). The results support the use of hip JPS tests in clinical practice and should be incorporated in assessing and managing elderly participants with hip OA.

## Introduction

Proprioception, according to Sherrington, is the perception of the position, motion of joints, and the perception of force in space^1^. Integrated action of different mechanoreceptors present in muscles, tendons, joint capsules, ligaments contributes to proprioception^2,3^. The proprioception is crucial in maintaining balance and body posture with precise and coordinated movements^4,5^. Proprioception is more important in older adults, especially regarding falls^1,6^. Aging affects proprioception, which affects awareness of body position in space^6^. Impaired position sense can significantly affect neuromuscular control and joint biomechanics, causing imbalance and increased risk of falls^1,7^. Previous research shows that decreased proprioceptive function can influence motor coordination and balance^6–12^.

Osteoarthritis (OA) primarily affects the lower extremities, causing functional disability^13^. Hip joint OA contributes to significant functional limitation and disability in the elderly^14^. Impaired proprioception may be a factor that initiates or progresses the degenerative changes in the joint^15^. Previous research has shown an increased likelihood of developing OA in the contralateral side in subjects with unilateral disease^16,17^. In addition, several studies have shown that people with hip OA have impaired joint position sense (JPS)^18^. Thus, proprioceptive accuracy may play a crucial role in hip OA.

The manner and type of evaluation used by therapists, clinicians, and institutions will differ according to considerations such as time constraints, the clinician's educational background, the availability of technology, and the specific movement or tissue being assessed. Different methods of testing hip JPS have been considered in current clinical practice, but the therapists must have access to a reliable tool that can accurately measure JPS^19^. Reproducibility involves how repeated tests using the same procedure in a study are obtained with the same findings^20^. Although the hip JPS is measured either in lying or in standing positions^21–23^, it is essential to find the most reliable position to evaluate the hip joint JPS. However, the reproducibility of these methods has rarely been investigated.

In the literature, different authors used devices to measure position sense such as electromagnetic tracking systems, Biodex systems, smartphone bubble inclinometer, and motion analysis systems and have reported good reliability (ICC = 0.75–0.89)^20,24–29^. In contrast, a few instruments, such as the modified goniometer and electro goniometer, demonstrated a low to moderate level of reliability (ICC = − 0.31 to 0.51)^30,31^. Furthermore, expensive and sophisticated equipment is challenging to operate in a clinical setting. On the other hand, the digital inclinometer is a simple device to use, affordable, requires less space, is managed by one rater, and makes quick measurements compared to expensive and sophisticated equipment^20,32^. However, given the lack of data on the reliability of inclinometer measurements of hip JPS, additional research is vital to equip clinicians and researchers with the information necessary to make clinical judgments regarding the measurement's accuracy. Therefore, this study aims to assess the intra-rater and inter-rater reliability of hip joint JPS tests using a digital inclinometer in elderly participants with unilateral hip osteoarthritis.

## Methods

### Design

The reliability study was conducted between January 2019 and December 2020 in the physical therapy department, King Khalid University, Kingdom of Saudi Arabia. This protocol consisted of planning, training, and agreement phases. Two examiners (examiner A and B) planned the hip JPS tests, repeated throughout the training period. Both the examiners agreed on the study procedures and standardized each test before actual testing.

### Subjects

Sixty-two patients (mean age: 67.5 ± 4.7 years) with a diagnosis of unilateral hip OA were referred to the rehabilitation department by a general physician or orthopedic doctor. Subjects with unilateral hip OA were included in the study if: they were > 50 years, hip flexion was < 115 degrees, and the diagnosis of hip OA met the clinical guidelines recommended by the American College of Rheumatology^33^. The participants were excluded if they had neurological disorders, had recent surgeries to the lower extremities, could not follow commands, and presented with any pre-existing comorbidities that would affect the testing. The participants did not take any medication for their hip pain. Before JPS testing sessions, all participants were told not to indulge in strenuous activities and continue their everyday lives. This study followed the Helsinki Declaration's guidelines, and the research ethics committee board of King Khalid University reviewed and approved this study (ECM#2021-4404). All the study participants signed informed consent before the commencement of the study.

### Examiners

The examiners who performed hip JPS tests and collected the study outcomes had experience in physical therapy musculoskeletal examination for more than ten years. Two recorders assisted the two examiners in study methods, and data collection; examiner A, recorder 1, and examiner B and recorder two were paired together. Examiners were blinded to each other’s assessment findings and data recordings. The intra-rater reliability was confirmed using assessments that were conducted on two different days with a gap of two days between the first and second. Comparing the assessments of examiners A and B on both the first and second evaluation sessions allowed us to determine their inter-rater reliability.

### Outcome measures

After completing the demographic assessment and questionnaires, participants performed hip JPS testings either with examiner A or B. A 15 min break was provided between two examinations, and each examiner's JPS testing session lasted for 15 min. All participants practiced and got familiarized with the hip JPS testing protocols before the actual testing.

### Measurement of hip joint position sense

**Figure 1:**
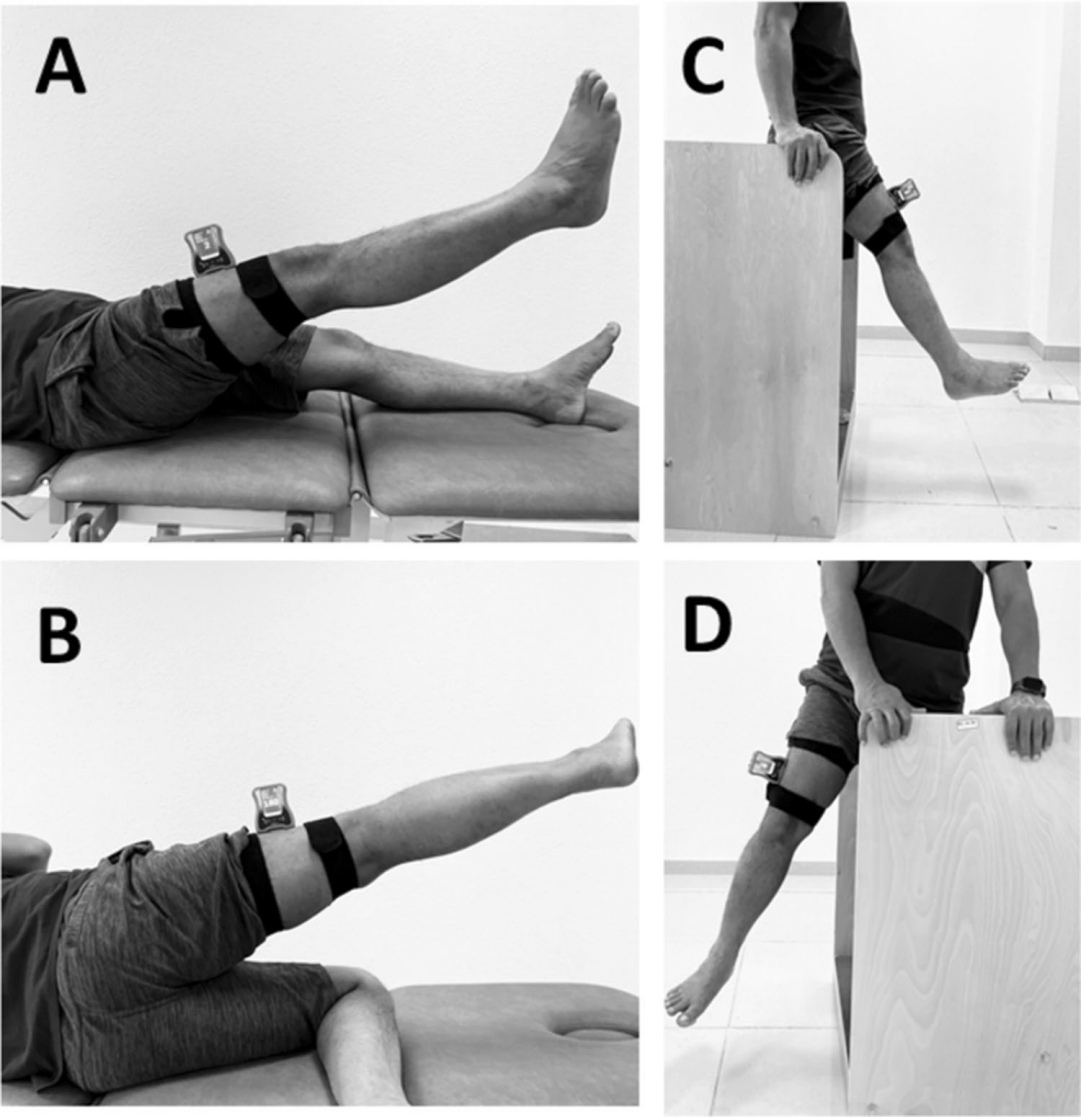
Hip joint position testing in (**A**) supine—hip flexion, (**B**) side-lying-hip abduction, (**C**) standing–hip flexion, (**D**) standing—hip abduction.

A digital inclinometer unit (Jtech Medical Industries, Inc.Salt Lake City, Utah) was used to evaluate hip joint JPS (Fig. 1). The tests were performed in a calm and quiet environment. All participants were checked on the affected side’ (diagnosed with unilateral hip OA). To test hip JPS in flexion, the digital inclinometer was positioned on the anterior and middle of the individual’s thigh and secured using a hook-and-loop strap. To test hip JPS in abduction, the inclinometer was placed on the lateral and middle aspects of the participant's thigh. All participants' hip full range of motion (ROM) was measured in both flexion and abduction directions, and 50% of their ROM was chosen as the target position during hip JPS testing. The JPS testing was performed in lying and in standing positions. All the subjects were blindfolded during the testing procedures.

To test hip JPS in a lying position, the participants were lying supine on the couch to test hip flexion and side-lying to test hip abduction. The digital inclinometer was strapped to the front of the thigh when testing JPS in hip flexion and lateral aspect of the thigh when testing for JPS in hip abduction (Fig. 1A, B). First, the participant's eyes were blindfolded; the affected limb (OA hip side) was tested for flexion and abduction using the “active-active" reproduction technique. Next, the examiner instructed the participants to flex or abduct their hip into the target position (50% of available ROM) and was asked to “stop” and maintain in this target position for five seconds and then to memorize this position. Following this, the participant's hip was guided back to the starting position. After this, the participants actively repositioned their hip to the target position, which was indicated by the participant saying “Yes.” Finally, the absolute difference between target and reproduced angle (joint position error) in degrees was considered to measure JPS accuracy^11,34–36^.

To test hip JPS in a standing position, the participants were asked to stand on a 15 cm high step with their non-testing side while the testing leg was provided complete freedom to flex or abduct at the hip (Fig. 1 C, D). Throughout the testing procedure in standing, participants were blindfolded and required to hold a wooden frame at hip height for added support. The "active-active" reproduction technique was followed as described above to assess the reposition accuracy in the standing position.

Each test in lying and standing was repeated three times, an average of the three scores was used for analysis. The order of JPS testing, either in lying or standing position and flexion or abduction direction, was decided by flipping a coin.

### Statistical analysis

SPSS Shapiro–Wilk Test was used to analyze if the study variables followed a normal distribution. The intraclass correlation coefficient (ICC (2. k), an absolute agreement, was used to assess the reliability^37^. As a guideline-recommended by Landis et al.^38^, the ICC values were interpreted as follows: > 0.80 was considered very good, 0.61–0.80 was considered good, 0.41–0.60 was considered moderate, 0.21–0.40 was considered fair, and 0.21 was considered poor. The limits of agreement (LOA) model was used to assess agreement between raters' scores^39^. Standard error of measurement (SEM) was recommended as the measure of agreement, and it was computed using the formula: $SD \times \sqrt 1 - {\text{ICC}}$. SD denotes the standard deviation. The minimal detectable change (MDC) was used to determine the minimum magnitude of change required to be 95% confident that the observed difference between the two tests was due to actual change rather than measurement error. 1.96 × SEM × √2 was used to determine the MDC^40^. The SPSS software (IBM, Chicago, IL, USA, version 24) was used to analyze the data of this cross-sectional study). A *p*-value of ≤ 0.05 was considered statistically significant to the study findings.

### Ethics approval

The Declaration of Helsinki guidelines were followed as a statement of ethical principles for medical research involving human participants. The work was approved by the King Khalid University Ethics and Research Committee (ECM#2021-4404).

### Consent to participate/consent to publish

All the participants signed an informed consent form prior to the commencement of the study. In addition, the participant signed informed consent to publish the image in an online open-access publication.

## Results

This study included 62 participants with a diagnosis of unilateral hip OA, completed two reliability assessment sessions (first and second) with examiners A and B. Table 1 summarizes the demographic characteristics of the study population.

**Table 1:** Demographic characteristics of patients with hip OA individuals.

Variable	Hip OA patients (n = 62)
Age (years)	67.5 ± 4.7
BMI (Kg/m^2^)	26.6 ± 3.5
VAS pain score (0–100 mm)	4.8 ± 1.0
HOOS (0–100 score)	61.4 ± 10.0
Supine-JPE in Hip Flexion (°) (mean ± SD)	3.86 (0.62)
Standing-JPE in Hip Flexion (°) (mean ± SD)	4.35 (0.50)
Side-lying-JPE in Hip Abduction (°) (mean ± SD)	3.96 (0.59)
Standing-JPE in Hip Abduction (°) (mean ± SD)	4.30 (0.40)

*BMI* body mass index, *VAS* visual analogue scale, *HOOS* hip disability and osteoarthritis outcome score, *JPE* joint position error, *SD* standard deviation.

### Intra-rater reliability

**Figure 2:**
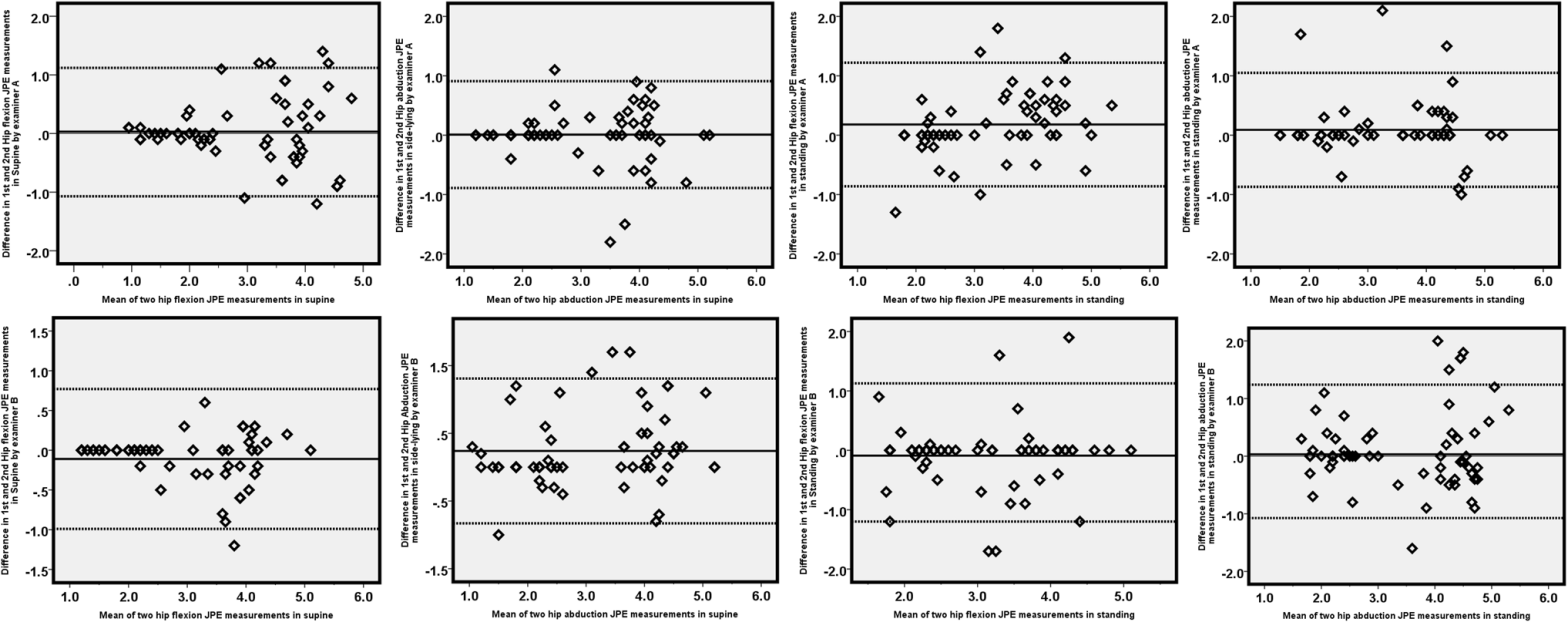
Bland–Altman plots of intra-rater reliability for hip flexion and abduction measurements by examiners A and B. The solid lines indicate the reference mean. The dotted lines indicate the upper and lower limits of agreement.

Table 2 summarizes the intra-rater reliability of hip JPS tests in the lying and standing positions. Figure 2 shows the Bland–Altman plots of mean and LOA for both examiners. Intra-rater reliability of hip JPS tests in the lying (supine/side-lying) position showed *very good* agreement in the directions of flexion (ICC: 0.88–0.92) and abduction (ICC: 0.89–0.91). Intra-rater reliability for hip JPS tests in the standing position was *good* in flexion (ICC: 0.69–0.72) and abduction (ICC: 0.66–0.69) directions.

**Table 2:** Intra-rater reliability of hip joint position tests (n = 62).

	ICC (Reliability)	95% CI	Mean diff AB (SD diff AB)	SEM	L.O. A	MDC
**Examiner A**						
Supine-JPE in hip flexion (°)	0.88	0.90–0.96	0.03 (0.56)	0.19	− 1.07 to 1.12	0.53
Standing-JPE in hip flexion (°)	0.69	0.57–0.82	0.18 (0.53)	0.29	− 0.86 to 1.22	0.80
Supine-JPE in hip abduction (°)	0.91	0.86–0.95	0.01 (0.46)	0.14	− 0.89 to 0.91	0.39
Standing-JPE in hip abduction (°)	0.69	0.63–0.86	0.09 (0.49)	0.27	− 0.87 to 1.05	0.75
**Examiner B**						
Supine-JPE in hip flexion (°)	0.92	0.92–0.97	− 0.11 (0.45)	0.13	− 0.99 to 0.77	0.36
Standing-JPE in hip flexion (°)	0.72	0.63–0.79	− 0.09 (0.57)	0.29	− 1.20 to 1.02	0.80
Supine-JPE in hip abduction (°)	0.89	0.78–0.93	0.24 (0.55)	0.18	− 0.83 to 1.31	0.50
Standing-JPE in hip abduction (°)	0.66	0.58–0.72	0.03 (0.62)	0.36	− 1.18 to 1.24	0.99

*JPE* joint position error, *95% CI* 95% confidence interval, *ICC agreement* Intraclass correlation coefficients, *Mean diff AB* mean difference between examiner A and B, *SD diff AB* standard deviation of the mean difference between day 1 and day 2, *SEM* standard error of measurement, *LOA* Limits of agreement, *MDC* minimal detectable change.

Examiner A showed the highest intra-rater reliability in hip abduction direction in lying position with ICC of 0.91 (95% CI = 0.86–0.95) and LOA between − 0.89 and 0.91. Likewise, Examiner B reported the most significant ICC values for hip flexion in the lying position with ICC of 0.92 (95% CI = 0.92–0.97) and LOA between − 0.99 to 0.77. The MDC varied from 0.36 to 0.80 degrees (lying and standing- hip flexion). In comparison, SEM ranged from 0.06 degrees (lying hip flexion and abduction, standing hip abduction) to 0.08 degrees (standing-hip abduction), and the MDC varied from 0.39 to 0.99 degrees (lying and standing-hip abduction) (Table 2).

### Inter-rater reliability

**Figure 3:**
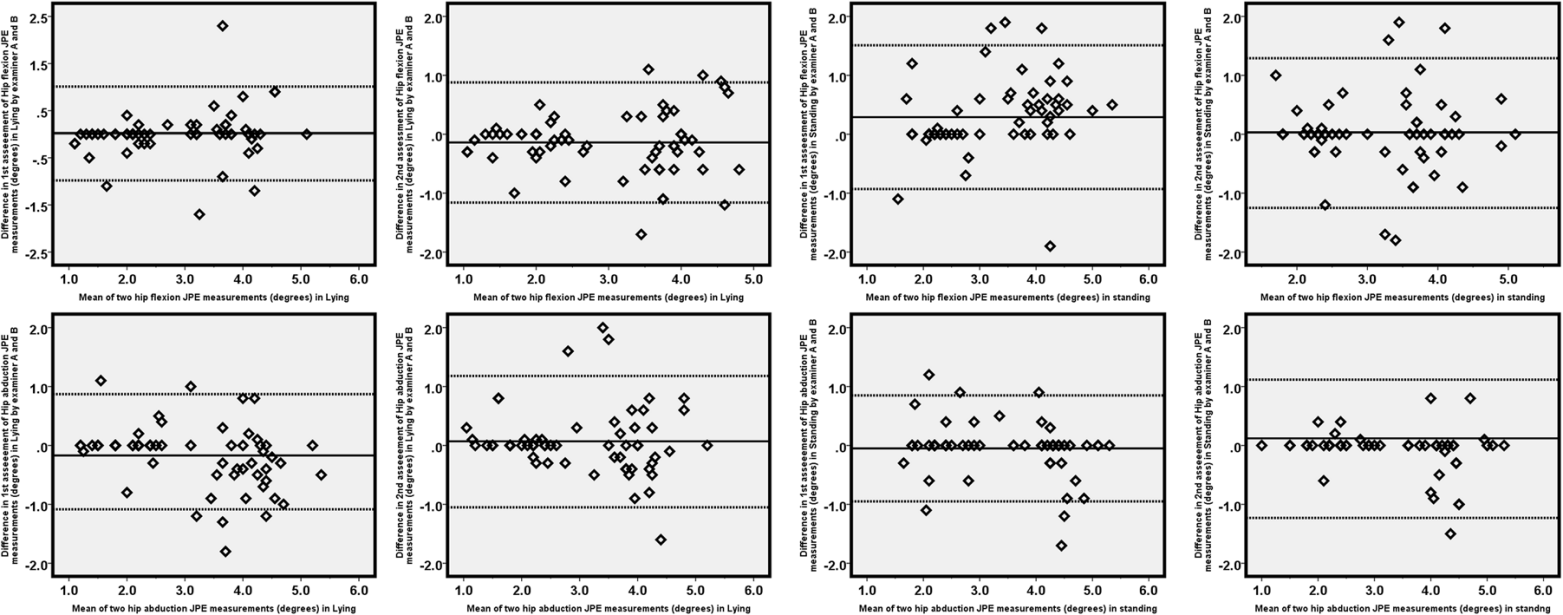
Bland–Altman plots of inter-rater reliability for hip flexion and abduction measurements by examiners A and B. The solid lines indicate the reference mean. The dotted lines indicate the upper and lower limits of agreement.

Table 3 summarizes the inter-rater reliability data. Inter-rater reliability for hip JPS tests generally ranged between good and *very good*, with ICC values ranging from 0.60 to 0.92. Figure 3 shows Bland–Altman plots with mean and LOA for the first and second evaluations. In the first assessment, *good* reliability was found for hip flexion in lying with an ICC of 0.64 (95% CI = 0.54–0.78) and good reliability for hip flexion in a standing position with an ICC of 0.89 (95% CI = 0.83–0.94). Similarly, *very good* reliability was noticed for hip abduction in lying with an ICC of 0.91 (95% CI = 0.82–0.93), and in the standing position, *good* agreement was exhibited with an ICC of 0.72 (95% CI = 0.66–0.84). The MDC’s ranged from 0.47 to 1.02 degrees in flexion direction and 0.44 to 0.66 degrees in hip abduction directions (Table 3).

**Table 3:** Inter-rater reliability of hip joint position tests (n = 62).

	ICC (Reliability)	95% CI	Mean diff AB (SD diff AB)	SEM	L. O. A	MDC
**First assessment**						
Supine-JPE in hip flexion (°)	0.89	0.83–0.94	0.01 (0.51)	0.17	− 0.98 to 1.01	0.47
Standing-JPE in hip flexion (°)	0.64	0.54–0.78	0.29 (0.62)	0.37	− 0.93 to 1.51	1.02
Supine-JPE in hip abduction (°)	0.91	0.82–0.93	− 0.17 (0.53)	0.16	− 1.21 to 0.87	0.44
Standing-JPE in hip abduction (°)	0.72	0.66–0.84	− 0.05 (0.46)	0.24	− 0.95 to 0.85	0.66
**Second assessment**						
Supine-JPE in Hip flexion (°)	0.87	0.82–0.93	− 0.14 (0.52)	0.19	− 1.16 to 0.88	0.53
Standing-JPE in hip flexion (°)	0.66	0.54–0.75	0.02 (0.65)	0.38	− 1.25 to 1.29	1.05
Supine-JPE in hip abduction (°)	0.87	0.80–0.92	0.07 (0.57)	0.20	− 1.05 to 1.18	0.55
Standing-JPE in hip abduction (°)	0.60	0.63–0.73	0.12 (0.69)	0.43	− 1.23 to 1.47	1.19

*JPE* joint position error, *95% CI* 95% confidence interval, *ICC agreement* Intraclass correlation coefficients, *Mean diff AB* mean difference between examiner A and B, *SD diff AB* standard deviation of the mean difference between examiner A and B, *SEM* standard error of measurement, *LOA* Limits of agreement, *MDC* Minimal detectable change.

In the second assessment, examiners found *very good* agreement in hip flexion direction in the lying position with an ICC of 0.87 (95% CI = 0.82–0.93) (Table 3). Also, the examiners found *good* agreement in hip flexion direction in the standing position with an ICC of 0.66 (95% CI = 0.54–0.75). The MDCs ranged between 0.47 and 1.05 degrees. The average discrepancies between examiners ranged from 0.01 degrees (SD = 0.51) to 0.29 degrees (SD = 0.62) (Table 3). Likewise, *very good* reliability was noted in the hip abduction direction (lying) with an ICC of 0.87 (95% CI = 0.80–0.92). A moderate agreement was demonstrated in the standing position with an ICC of 0.60 (95% CI = 0.63–0.73). The MDC’s ranged from 0.53 to 1.05 degrees in flexion direction and 0.55 to 1.19 degrees in hip abduction directions (Table 3).

## Discussion

The study aimed to evaluate hip JPS tests using a simple and affordable digital inclinometer in the lying and standing positions. The findings of this study demonstrated promising intra-rater and inter-rater reliability for assessing hip JPS in elderly individuals with unilateral hip OA.

This study’s reliability (intra- and inter-rater) data were good, with ICC, MDC, and SEM values. In addition, there was no consistent bias between the examiners, as shown in Bland Altman LOA plots. In the literature, only a few studies used ICC, SEM, and MDC to establish hip JPS test reliability in lying and standing. This study’s results are in accordance with Benjaminse et al.^24^, who demonstrated *good intra-rater* reliability for hip JPS in healthy individuals (age range: 18 and 30 years) with an ICC of 0.753 and SEM of 0.248 degrees. Hip JPS was evaluated using Biodex System 3 and a Vicon Motion Analysis System. In the flexion direction, they showed an ICC of 0.76 (SEM = 0.26°), and in the abduction direction, they demonstrated an ICC of 0.26 (SEM = 0.26°)^24^.

Contrary to our results, Arvin et al.^41^ showed poor reliability for hip abduction JPS assessed in a standing position (ICC: 0.11, SEM: 0.39 degrees, and LOA: 0.54 degrees to 2.08 degrees) 19 healthy older individuals^41^. In Arvin et al. study, the hip JPS was assessed in 19 healthy older individuals using the Optotrak system. However, the results of Arvin et al. can’t be compared to the conclusions of this study due to methodological differences. The hip JPS test positions in Arvin et al. study was assessed using the Optotrak system, and each participant performed 24 trials and a 10-min rest period between JPS tests. Therefore, excessive attempts may have produced fatigue or lowered participants' concentration, affecting the study’s reliability^41^. The authors also discussed that the poor findings could be related to the difficulty of maintaining balance in a single-legged standing stance with eyes closed.

Different authors have reported that an inclinometer is a reliable tool for assessing hip JPS. Baert et al.^42^ demonstrated good to excellent reliability (intra-rater and inter-rater) of knee JPS tests measured using an analogue inclinometer in asymptomatic (ICC = 0.65–0.85) and knee OA patients (ICC = 0.70–0.95)^42^. Romero-Franco et al.^43^ demonstrated that an inclinometer is a valid (ICC = 1.0, p0.001) and reliable (ICC = 1.0, p0.001) tool for measuring knee joint position sensing in closed kinetic chain positions. Alahmari et al. ^20^ evaluated the reliability of neutral head and target head reposition sensibility in subjects with and without neck pain using a digital inclinometer and found the tool reliable. Barrett et al.^44^ showed *very good* intra-rater (ICC = 0.94) and inter-rater (ICC = 0.86) reliability of measuring spinal curves using an inclinometer.

In this study, compared to standing, the lying (supine and side-lying) position demonstrated superior reliability in hip JPS testing. This finding could be due to the stability and large base of support provided by the lying (supine and side-lying) position during the testing^45^. The standing position is more challenging to maintain balance when tested on a single -leg with eyes closed^45^. The participants may have difficulty focusing on the hip JPS tests while multitasking. This study assessed hip JPS in the open kinetic chain positions. It may be worth considering JPS testing in the standing position to reflect the more functional position. The lying (supine and side-lying) position can be considered for weak individuals and patients presenting with increased pain not allowing the standing position to test hip JPS. The individuals have extra support and can concentrate more effectively on the hip joint to maintain balance, which might have improved the consistency of trials. JPS testing in weight-bearing positions was also considered by different authors^46,47^, as weight-bearing tests are more functional and involve all cutaneous, articular, and muscle proprioceptors that work together during everyday activities^46^. The disadvantage of testing JPS in a weight-bearing position is that you cannot exclude proprioceptive information from the knee or ankle joints. So deficits in hip JPS can be masked by efficient proprioceptive abilities in the knee and ankle joint.

The LOA can be used to determine whether an individual's performance is "real." For example, suppose the difference between the two measurements is more significant than the LOA. In that case, the difference is likely the result of the intervention, indicating an actual change in the individual's performance, independent of measurement error^48^. Our study's LOA interval was relatively small compared to earlier studies that evaluated hip JPS^49,50^. This finding demonstrates that our test can detect subtle changes in an individual's hip proprioception over time.

## Limitations

Hip JPS was measured in this study using a digital inclinometer. Although every attempt was made to limit cutaneous feedback to a minimal level during JPS testing, friction between the gluteal region and cutaneous input from the couch might have influenced this study's results. Because all participants wore spandex shorts, measures were made to ensure that friction force and clothing folding were consistent across the study. Little research is available on the effect of cutaneous input from clothing or surrounding structures on proprioceptive sense. Standing position, leg movement velocity, environmental conditions, and participants' focus may have influenced our test's reliability. However, the reliability was found good in this study, and this is most likely because the study protocols and methods were consistent, allowing the examiners to collect the outcome accurately.

## Conclusion

This study demonstrated good reliability for hip JPS using a digital inclinometer in subjects with unilateral hip OA. In addition, the lying position showed superior hip JPS reliability than the standing position. Therapists should consider the MDC values when interpreting change values obtained during subsequent measurement sessions to ensure that the change is not attributable to intertrial variability or measurement error. However, further research is needed to determine the test's validity and discriminative ability in various hip pain populations and look into its diagnostic accuracy.

## Data Availability

On request to the corresponding author Ravi Shankar Reddy (rshankar@kku.edu.sa), all data are available in the medical rehabilitation sciences.
